# Booster Immunization Improves Memory B Cell Responses in Older Adults Unresponsive to Primary SARS-CoV-2 Immunization

**DOI:** 10.3390/vaccines11071196

**Published:** 2023-07-04

**Authors:** Marije K. Verheul, Kim H. Nijhof, Mary-lène de Zeeuw-Brouwer, Geraly Duijm, Hinke ten Hulscher, Lia de Rond, Lisa Beckers, Dirk Eggink, Sophie van Tol, Johan Reimerink, Mardi Boer, Josine van Beek, Nynke Rots, Rob van Binnendijk, Anne-Marie Buisman

**Affiliations:** 1 Centre for Immunology of Infectious Diseases and Vaccines, Center for Infectious Disease Control, National Institute for Public Health and the Environment, 3721 MA Bilthoven, The Netherlands; 2 Centre for Infectious Diseases Research, Diagnostics and Laboratory Surveillance, WHO COVID-19 Reference Laboratory, Center for Infectious Disease Control, National Institute for Public Health and the Environment (RIVM), 3721 MA Bilthoven, The Netherlands

**Keywords:** memory B cells, SARS-CoV-2, older adults, coronavirus, booster, primary vaccination, antibodies

## Abstract

The generation of a specific long-term immune response to SARS-CoV-2 is considered important for protection against COVID-19 infection and disease. Memory B cells, responsible for the generation of antibody-producing plasmablasts upon a new antigen encounter, play an important role in this process. Therefore, the induction of memory B cell responses after primary and booster SARS-CoV-2 immunizations was investigated in the general population with an emphasis on older adults. Participants, 20–99 years of age, due to receive the mRNA-1273 or BNT162b2 SARS-CoV-2 vaccine were included in the current study. Specific memory B cells were determined by ex vivo ELISpot assays. In a subset of participants, antibody levels, avidity, and virus neutralization capacity were compared to memory B cell responses. Memory B cells specific for both Spike S1 and receptor-binding domain (RBD) were detected in the majority of participants following the primary immunization series. However, a proportion of predominantly older adults showed low frequencies of specific memory B cells. Booster vaccination resulted in a large increase in the frequencies of S1- and RBD-specific memory B cells also for those in which low memory B cell frequencies were detected after the primary series. These data show that booster immunization is important for the generation of a memory B cell response, as a subset of older adults shows a suboptimal response to the primary SARS-CoV-2 immunization series. It is anticipated that these memory B cells will play a significant role in the immune response following viral re-exposure.

## 1. Introduction

It was estimated that severe acute respiratory syndrome coronavirus 2 (SARS-CoV-2) was responsible for over 3 billion infections worldwide [1] and over 6 million deaths before the rise of the Omicron variants (World Health Organization (WHO), 1 July 2022). To prevent excess mortality and increased hospitalization, over 11 billion doses of vaccine have been distributed (WHO, 1 July 2022). Most of these are messenger ribonucleic acid (mRNA) vaccines (BNT162b2 (Comirnaty), mRNA-1273 (Spikevax)), vector-based vaccines (ChAdOx1 nCov-19 (Vaxzevria), Ad.26.COV2.S (Janssen)), or inactivated/live-attenuated vaccines (CoronaVac)). In Europe, many people have now received a primary immunization series and at least one booster immunization, depending on local policies. Further immunization rounds are often under consideration, as initial protection induced by immunization seems short-lived or insufficient in vulnerable populations [2]. In addition, this is first time that mRNA vaccines have been used on such a large scale and further understanding of the induced immune response may assist in the development of future mRNA vaccines. It is, therefore, important to evaluate the immune response induced by primary and booster immunizations, especially in population groups more vulnerable for serious disease, such as older individuals.

Although it is not fully known which parts of the immune system after vaccination or infection with SARS-CoV-2 are responsible for protection against infection or severe disease, it has been suggested that antibodies play an important role, as these are often found to correlate with protection or vaccine efficacy [3,4]. Studies on monoclonal antibody therapy or antibody transfer efficacy indicate that there is a role for the humoral immune response in protection against SARS-CoV-2 infection [5,6,7]. However, antibody concentrations wane after vaccination, indicating that cellular immunity is likely important for long-term protection by sustaining antibody production over time [8,9,10]. Upon a next encounter with SARS-CoV-2 antigens, memory B cells will undergo rapid reactivation that accounts for a secondary antibody response. After reactivation, memory B cells residing in secondary and tertiary lymphoid tissues recirculate and are able to differentiate into antibody-secreting short-lived plasma cells, long-lived plasma cells, or renewed memory B cells [11,12]. Whether a good response can be induced is dependent on many factors, such as antigen presentation, the induction of T cell immunity, and the number of specific naïve or memory B cells available [13].

Generally, the age-related decline in immune function, referred to as immunosenescence, contributes to reduced vaccine efficacy in older adults, as documented for influenzavirus, for example. In addition, the antigen-specific memory B cell recall response is lower in older adults as aged memory B cells are less capable of differentiating into antibody-producing plasma cells upon boosting [14,15,16]. Responsiveness to a new antigen will be dependent on the availability and function of naïve B and T cells, which are influenced by genetics, age, and several environmental factors, among others [17,18]. In this respect, and in contrast to the influenza virus, the introduction of a new virus such as SARS-CoV-2 provides a unique opportunity to evaluate the immune responsiveness at first antigen encounter and subsequent booster immunization in (older) adults.

Vaccine efficacy against infection with the SARS-CoV-2 delta variant after the primary immunization series was reduced in older adults compared to younger age groups [19]. A further reduction in vaccine effectiveness against hospitalization or symptomatic disease was noted after the introduction of Omicron variants [20,21]. However, differences between variants and age groups are difficult to establish due to potential confounding factors, including exposure frequency and time since last immunization. As some older adults are also more prone to a more severe outcome after SARS-CoV-2 infection, it is important that differences in immune response between young and older adults are understood. Therefore, we investigated the induction of SARS-CoV-2 memory B cell responses after primary and booster mRNA immunizations in participants aged 20–99. 

## 2. Materials and Methods

### 2.1. Study Design and Participant Selection

Data collected from multiple SARS-CoV-2 immunization cohorts were combined for the current analysis of B-cell responses post mRNA vaccination. Two studies were prospective observational studies, recruiting participants planning to receive a SARS-CoV-2 immunization. The first study recruited older participants aged 64–90, who previously participated in the immune system and ageing (ISA) substudy of the Doetinchem Cohort Study (DCS, EudraCT: 2021-002363-22) [22,23]. The second study recruited participants aged up to 60 years, by approaching people from the general population (NL76440.041.21, EudraCT: 2021-001357-31) [24]. The third study is the ongoing longitudinal intervention study Vital, originally designed to study influenza and pneumococcal conjugate vaccines (EudraCT Number: 2019-000836-24) [25]. The institutional Review Board (or Ethics Committee) of the Medical Research Ethics Committee Utrecht approved all three studies. The studies were carried out in accordance with the declaration of Helsinki and informed consent was obtained from all study participants.

Within the first two observational studies, participants received an mRNA vaccine (BNT162b2 (Comirnaty) or mRNA-1273 (Spikevax)) through the national immunization program. Within the third study, participants received a SARS-CoV-2 immunization (mRNA-1273 (Spikevax)) as part of the study. Participants were sampled and filled in questionnaires before and at 28 days after completion of the primary immunization series (P28), before administration of a booster immunization (B0), and 28 days after the booster immunization (B28). Participants with a history of infection based on a positive test or the presence of SARS-CoV-2-specific antibodies (Spike S1 or nucleoprotein) were excluded from the current analysis. An overview of participant characteristics can be found in Table 1. 

### 2.2. Processing of Serum and PBMC Samples

Blood sample tubes (2.7 mL tubes, 04.1923.001, Sarstedt, Nümbrecht, Germany) were spun down at 2500× *g* for 10 min or blood sample tubes (Vacuette 8 mL tubes, 485502, Greiner Bio-one, Alphen aan den Rijn, the Netherlands) were spun down at 1800× *g* for 10 min. Serum was aliquoted and frozen at −80 °C until use.

Peripheral blood mononuclear cells (PBMCs) were collected from heparine tubes and initially spun down for 10 min at 700× *g* for plasma collection. PBMCs were further collected by adding Lymphoprep (1114547, Progen, Heidelberg, Germany), spinning down at 800× *g* for 30 min with a low break. Cells were washed at least twice with PBS/0.2%FBS (phosphate-buffered saline/fetal bovine serum from Hyclone, GE Healthcare, Chicago, Illinois, USA, heat inactivated at 56 °C for 30 min). After counting with a DxH500 hematology analyzer (Beckman Coulter, Woerden, the Netherlands), cells were frozen in FBS and 20% DMSO and stored at −135 °C until use. 

### 2.3. SARS-CoV-2 Antibody Multiplex Immunoassay

SARS-CoV-2 antibody levels in serum were determined using a multiplex immunoassay (MIA) as described previously [26]. In short, beads coupled to SARS-CoV-2 antigens Spike S1 (Sino Biological, Beijing, China 40591-V08H), receptor-binding domain (RBD, Sino Biological, 40592-V08B), or N (Sino Biological, 40588-V08B) were incubated with serum samples and antibody binding was detected with an Fm3D (Luminex, Austin, TX, USA). Antibody levels were normalized to binding antibody units (BAU)/mL. Antibody levels described in this paper were previously published for some of the cohorts [24].

### 2.4. SARS-CoV-2 Antibody Avidity Assay

To measure SARS-CoV-2 antibody avidity, adjustments were made to the standard SARS-CoV-2 MIA assay described above by adding one additional step. After incubation of the beads and serum samples to allow antibody binding, 0 M or 2 M of ammonium thiocyanate (Sigma, Amsterdam, The Netherlands) was added for 10 min before detecting the remaining binding antibodies. The avidity index (AI) is expressed as the percentage of residual mean fluorescence intensity (MFI) for the 2 M condition in comparison to the 0 M condition, which was set to 100%. 

### 2.5. SARS-CoV-2 Neutralization Assay

Sera neutralization capacity against the ancestral SARS-CoV-2 virus (German isolate; GISAID ID EPI_ISL 406862; European Virus Archive Global #026V-03883) was tested as previously described [27]. Briefly, samples were serially diluted in Dulbecco modified Eagle medium (DMEM) supplemented with NaHCO3, HEPES buffer, penicillin, streptomycin, and 1% fetal bovine serum, starting at a 1:10 dilution in 50 μL. Subsequently, 50 μL of virus suspension was added to each well and incubated at 35 °C for 1 h. Vero E6 cells were added in a concentration of 20,000 cells per well and subsequently incubated for 48 h at 35 °C. After incubation, cells were fixed with 4% formaldehyde/(PBS) and stained with a nucleocapsid targeting monoclonal antibody. Bound antibodies, as a measure for infected cells, were detected using horseradish peroxidase–conjugated goat anti-human IgG (1:3000) in 2% milk/PBS for 1 h at RT. After washing, the color reaction was developed using 3,3′,5,5′-tetramethylbenzidine substrate (Thermo Fisher, Waltham, MA, USA). The reaction was stopped by adding 0.8 N sulfuric acid, and OD450 (optical density at 450 nm) was measured using standard equipment. IC50 values were calculated using Graphad Prism.

### 2.6. SARS-CoV-2 Memory B Cell ELISpot

Frozen PBMC samples were thawed and cultured at 37 °C for 5 days in AIM V with AlbuMAX (Thermo Fisher, Waltham, MA, USA), supplemented with 10% heat-inactivated FBS and 50 µM β-mercapto-ethanol. The 96-well plate culture wells contained 200,000 cells and were polyclonally activated by 3 µg/mL CpG, 10 ng/mL recombinant Interleukin 2, and 10 ng/mL recombinant interleukin 10 diluted in culture medium. 

ELISpot filtration plates (MSIPS4510, Millipore, Darmstadt, Germany) were pre-wetted with 70% ethanol and coated with 10 ug/mL of SARS-CoV-2 S1 (Sino biological, 40591-V08H), RBD (Excellgene, Monthey, Switzerland), N (Sino Biological, 40588-V08B), S1 Omicron BA.1 (Sino biological, 40591-V08H), MERS S1 (Sino Biological, 40069-V08H), or goat anti-human IgG (855071, MP Biomedicals, Eschwege, Germany). All antigens were diluted in PBS, except N, which was diluted in carbonate buffer (C130.76.0200, Tritium Microbiology, Eindhoven, the Netherlands). PBS was added to the blank wells. The SARS-CoV-2 antigens were tested in duplicate at three different cell concentrations (200.000, 66.000, 22.000 cells/well), except for S1 Omicron BA.1, for which one replicate was tested for a subset of participants. The blank and MERS-S1 controls were tested at the highest cell concentration only (200.000 cells/well). Total IgG production was measured as a positive control, using 22,000, 2000, or 200 cells/well. Coated plates were stored up to 7 days at 4 °C.

Before adding cells, the ELISpot plates were washed 3 times with 200 uL PBS per well, and incubated with culture medium for at least 1 h at 37 °C. After blocking, previously cultured cells diluted in culture medium were added to the coated ELISpot plate and incubated for 18–20 h at 37 °C. 

ELISpot plates were washed 4 times with 200 µL/mL of washing buffer (PBS with 0.05% Tween20) and cells were lysed with 150 µL/well of milliQ for 5 min, after which the plates were washed another 4 times with washing buffer at 200 µL/mL. Antibody binding was detected with 0.2 µm filtered 50 µL/well AP-conjugated goat-anti-human IgG (5220-0462, Seracare, Milford, MA, USA), diluted in 1:5000 in PBS/0.01% Tween20/1% goat serum (ICN Biomedicals, 191356), and incubated for 1–2 h at 37 °C. After a total of eight washing steps with washing buffer, water, and PBS, spots were made visible by incubation with BCIP (B5655 Sigma, or KPL, Seracare, 5420-0038), incubated up to 15 min at room temperature. Spots were imaged and counted using the same settings throughout each experiment (Immunospot C.T.L, Sample sensitivity = 185, Spot separation = 5, gating = 0.0133 to 0.3339, diffuse processing = large). Data were normalized to the number of cells originally added to the well of the ELISpot plate. The cut-off for positivity was set at the mean plus three times the standard deviation of the MERS-S1 control.

### 2.7. Flow Cytometry

PBMC samples were investigated by flow cytometry both before and after 5 days of stimulation as described under the ELISpot section above. In addition, 100.000–200.000 cells per well were stained for lymphocytes with anti-human CD45-PE (555483, BD Biosciences, Eysins, Switzerland), for B cells with anti-human CD19-PerCPCy5.5 (BD Biosciences, 561295), for memory B cells with anti-human CD27-FITC (BD Biosciences, 555440), and for plasmablasts with anti-human CD38-APC (BD Biosciences, 555462), for 20–30 min at 4 °C. All were diluted in PBS/0.5% BSA/2 mM EDTA. Samples were incubated in fixation buffer (Biolegend, San Diego, CA, USA, 420801) for 20 min at 4 °C before acquisition on a FACSCanto (BD). Data were analyzed with FlowJo version 10.8 and gated as in Supplementary Figure S2. Single cells were gated with SSC-A, SSC-H, FSC-A, and FSC-H.

### 2.8. Statistics

Data were analyzed with R version 4.3.0. Differences between independent datasets were investigated with a Mann–Whitney U test. Paired data were compared with a paired Wilcoxon signed-rank test. Potential linear correlations were investigated with a Pearson correlation test. A *p*-value less than 0.05 was considered significant.

## 3. Results

### 3.1. A Subset of Adults Does Not Have Detectable Frequencies of Specific Memory B Cells after Completion of the Primary Vaccination Series

**Figure 1:**
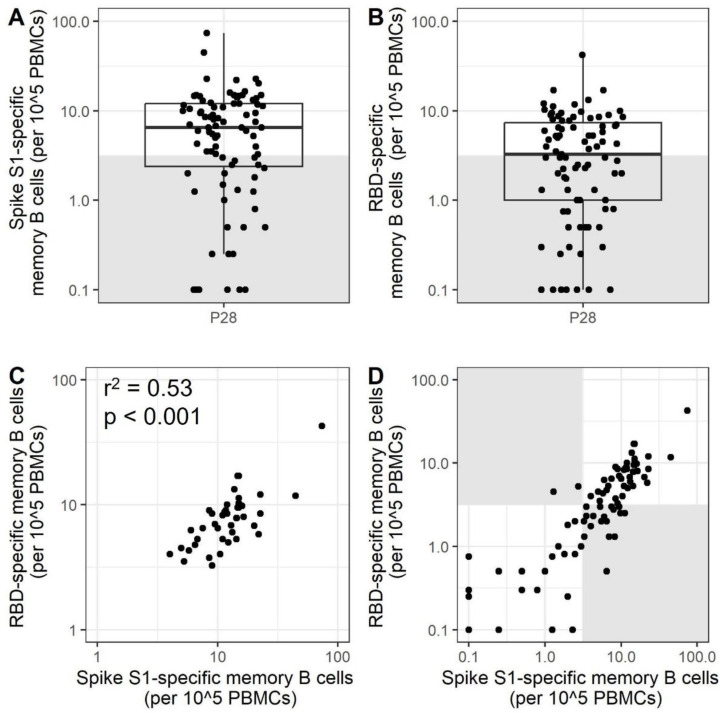
SARS-CoV-2 Spike S1- and RBD-specific memory B cells are present in a subset of vaccinees after the primary mRNA immunization series. SARS-CoV-2 Spike S1- (**A**) and RBD-specific (**B**) memory B cells were quantified by ELISpot (N = 87). Samples within the grey box are below the cut-off and are considered low-responders. The lower and upper hinges of the boxplot indicate the 25th and 75th percentile, with the middle line indicating the median. The whiskers extend up to 1.5 ×the inter-quartile range. A correlation between Spike S1- and RBD-specific spots at P28 is shown for participants with a response above the cut-off value for both RBD and Spike S1 (N = 42) (**C**). The strength of the correlation was estimated with a Pearson correlation. The correlation shown in (**D**) includes all participants in (A/B), where the grey boxes contain participants classified as a low-responder for one of the two antigens. PBMCs = peripheral blood mononuclear cells; RBD = receptor-binding domain; P28 = 28 days after completion of the primary immunization series.

To investigate the presence of SARS-CoV-2-specific memory B cells to S1 and RBD proteins after mRNA immunization, a total of 88 participants, with ages ranging from 20 to 99, were included in the current study (Table 1). The cut-off for a response was set at 3.1 based on the MERS-S1 controls (Figure S1) and participants with memory B cell frequencies below the cut-off will be referred to as low-responders. For 68.9% of the participants, measurable SARS-CoV-2 Spike S1-specific memory B cells were identified, while RBD-specific memory B cells were detected in 50.5% of the participants after the primary immunization series (Figure 1A,B). The number of specific B cells detected per 100.000 PBMCs was 6.5 (2.4–12) (median (IQR)) for S1 and 3.2 (1.0–7.4) for RBD. For participants with a detectable response for both antigens (48.2%), there is a correlation between the number of S1- and RBD-specific memory B cells (Figure 1C, *p* < 0.001 and R^2^ = 0.53). For a substantial number of participants (29%), no specific memory B cells were detected for Spike S1 nor for RBD. For 2.3% of the participants, a response towards RBD was observed but not for S1, while 20.6% of participants showed a response towards S1 but not RBD (Figure 1D). Importantly, these data indicate that a subset of adults do not have detectable SARS-CoV-2-specific memory B cells after the primary immunization series.

### 3.2. Older Adults Exhibit Less SARS-CoV-2-Specific Memory B Cells after Immunization

**Figure 2:**
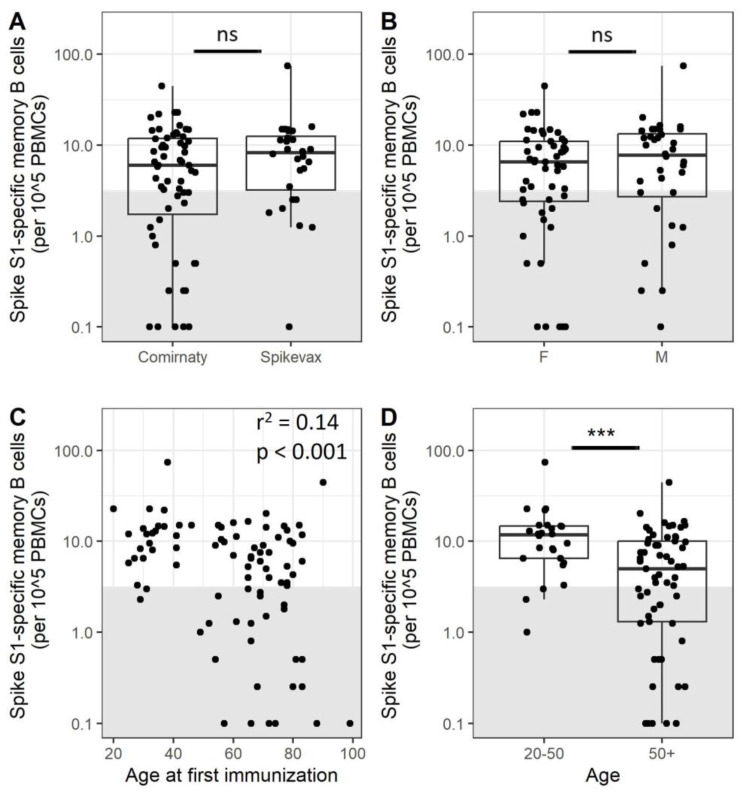
A subset of older adults does not generate a good memory B cell response after the SARS-CoV-2 primary immunization series. The SARS-CoV-2 Spike S1 memory B cell response 28 days after completion of the primary immunization series was compared between Comirnaty (N = 59) and Spikevax (N = 28) immunization (**A**), male (N = 35) or female (n = 52) participants (**B**), and between participants split into two different age groups (N = 26 for 20–50 and N = 61 for 50+) (**D**). Samples within the grey box are below the cut-off and are considered low-responders. The lower and upper hinges of the boxplot indicate the 25th and 75th percentile, with the middle line indicating the median. The whiskers extend up to 1.5 × the inter-quartile range. A potential correlation between SARS-CoV-2 Spike S1 memory B cells and age was further investigated (N = 87) (**C**). Groups were compared using a Mann–Whitney U test. Correlations were investigated using a Pearson correlation. PBMCs = peripheral blood mononuclear cells; ns = not significant; *** = *p* < 0.001.

We further investigated whether age, sex, and vaccine type contribute to the observed differences in B cell memory response measured by ELISpot. In this respect, there were no significant differences between participants depending on the mRNA vaccine received (*p* = 0.23 for S1, *p* = 0.10 for RBD, Figure 2A and Figure S3A) nor between male and female participants (*p* = 0.39 for S1, *p* = 0.45 for RBD, Figure 2B and Figure S3B). There is a weak correlation between age and S1 memory B cell frequencies (Figure 2C, *p* < 0.001 r^2^ = 0.14 for S1, see Figure S3C for RBD, *p* = 0.02, r^2^ = 0.07). In addition, the figure shows that a large number of low-responders was present in the older adult population. To further explore this, participants were split into two age groups above or below 50 years of age. The resulting data indicate that adults over 50 years of age have reduced S1 memory B cell frequencies compared to younger adults (Figure 2D, *p* < 0.001). Within the adults over 50 years of age, 39% were classified as low-responders, while this was 12% for the younger adults. A similar pattern can be observed for RBD-specific memory B cells (Figure S3D, *p* < 0.001). It should be noted that the percentage of total B cells as measured by flow cytometry was reduced in older adults compared to younger adults 5.9% vs. 9.7%, a difference that was further expanded after ex vivo proliferation and stimulation of PBMCs (Table S1). Polyclonal stimulation and expansion are required for the detection of specific memory B cells. However, these findings could also reflect a difference between older and younger adults in their B cells’ capacity to respond to stimuli. Phenotyping of PBMCs before and after proliferation showed expansion of B cells, memory B cells, and plasmablasts (Figure S2) and revealed a slightly lower percentage of plasmablasts (11.0% vs. 3.3%, *p* = 0.002) and memory B cells (15.6% vs. 5.43%, *p* = 0.004) present after proliferation for low-responders compared to responders (Figure S4). The previously observed difference in numbers of S1 memory B cells between age groups quantified by ELISpot becomes smaller after correcting for the number of plasmablasts or memory B cells per person identified by flow cytometry, but a group of low-responders remains (Figure S5). These data indicate that there is a subset of participants less responsive to SARS-CoV-2 immunization, mostly within the older adult population. The low memory B cell response is only partially explained by differences in B cell subset composition.

### 3.3. Participants without Memory B Cells Show Reduced Antibody Levels and Functionality

**Figure 3:**
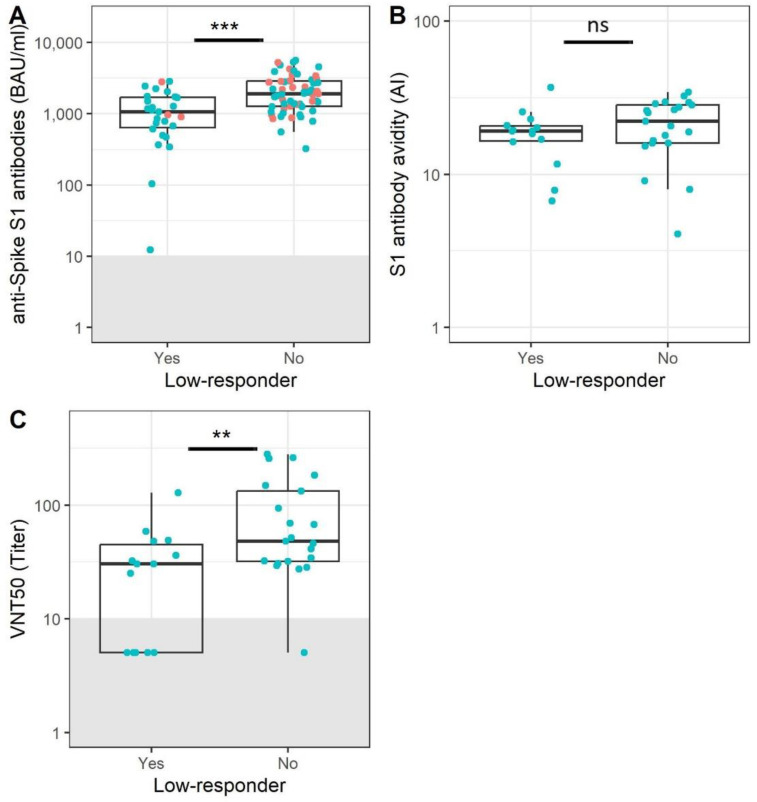
SARS-CoV-2 memory B cell non-responders show reduced antibody levels and functionality. Anti-Spike S1 antibody levels were compared between Spike S1 memory B cell non-responders (N = 27) and responders (N = 60) 28 days after completion of the primary immunization series (**A**). Samples within the grey box are below the cut-off for antibody positivity. Anti-Spike S1 antibody avidity was compared between Spike S1 memory B cell responders (N = 26) and non-responders (N = 14) 28 days after completion of the primary immunization series (**B**). Virus neutralization titers were compared between Spike S1 memory B cell non-responders (N = 15) and responders (N = 27) 28 days after completion of the primary immunization series (**C**). Samples within the grey box were below the lower limit of detection. All samples below the lower limit of detection were assigned a titer of 5. In both cases, a Mann–Whitney U test was used to investigate differences between groups. The lower and upper hinges of the boxplot indicate the 25th and 75th percentile, with the middle line indicating the median. The whiskers extend up to 1.5 × the inter-quartile range. Red dots indicate participants aged 20–50, while blue dots indicate participants over 50 years of age. AI = avidity index; BAU = binding antibody units; VNT = virus neutralization titer; *** = *p* < 0.001, ** = *p* < 0.01.

Although it was not possible to detect SARS-CoV-2-specific memory B cells in a subset of older adults 28 days after the first two vaccinations, all participants had measurable Spike S1 serum antibodies. However, memory B cell low-responders had significantly lower antibody levels compared to participants in which specific memory B cells were detected (Figure 3A, 9.7 BAU/mL vs. 95.9 BAU/mL, *p* < 0.001). To investigate whether antibody functionality could be compromised, both the virus-neutralizing capacity and binding strength (avidity) of the acquired antibodies were measured in a subset of participants. No differences in avidity were observed (Figure 3B, *p* = 0.3), but low-responders for memory B cells had lower antibody neutralization titers (Figure 3C, 30.4 vs. 48.2, *p* = 0.01). Overall, the level and quality of the antibody response to SARS-CoV-2 immunization was also lower in memory B cell low-responders. 

### 3.4. Memory B Cell Responses after Primary mRNA Vaccination Do Not Decrease during the First 5–9 Months after Vaccination

**Figure 4:**
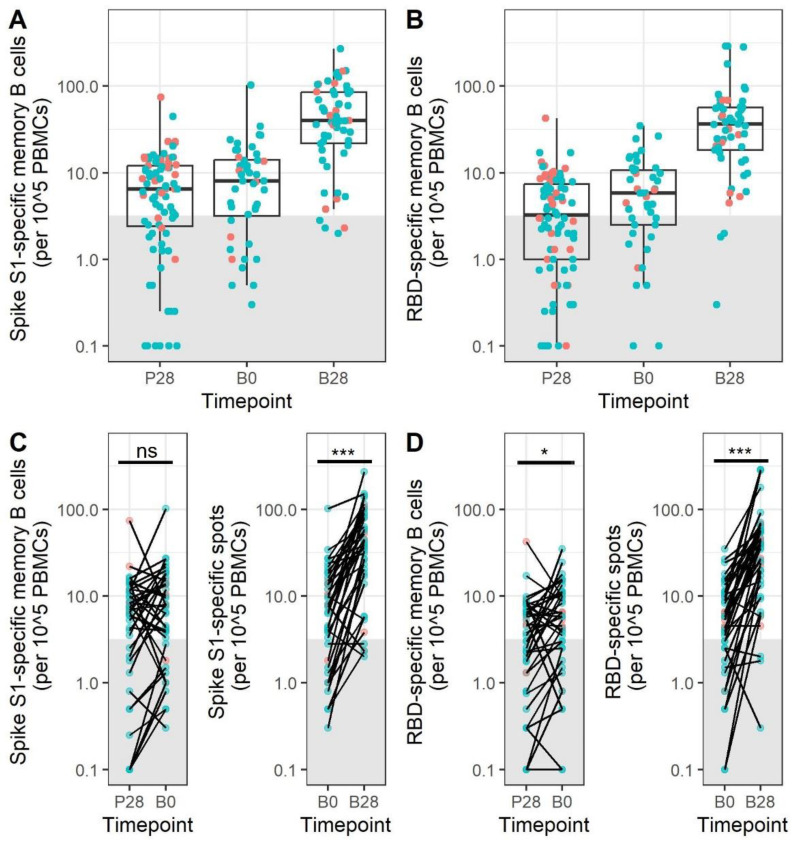
SARS-CoV-2 mRNA booster immunization increases the memory B cell response. SARS-CoV-2 Spike S1-specific (**A**) and RBD-specific (**B**) memory B cell responses are shown at different timepoints after mRNA immunization. N = 87, 44, and 58 at P28, B0, and B1, respectively. The lower and upper hinges of the boxplot indicate the 25th and 75th percentile, with the middle line indicating the median. The whiskers extend up to 1.5 × the inter-quartile range. Samples within the grey box are below the cut-off for positivity. Changes in memory B cell responses over time in paired samples are shown for SARS-CoV-2 Spike S1 (**C**) and RBD (**D**). N = 43 for samples paired between P28 and B0. N = 41 for samples paired between B0 and B1. Red dots indicate participants aged 20–50, while blue dots indicate participants over 50 years of age. Groups were compared with a paired Wilcoxon signed-rank test. P28 = 28 days after the primary immunization series; B0 = before booster immunization; B28 = 28 days after booster immunization; PBMCs = peripheral blood mononuclear cells; RBD = receptor-binding domain; ns = not significant, * *p* < 0.05, *** = *p* < 0.001.

To investigate whether memory B cell responses change over time, samples taken before booster immunization were measured (B0). The time between the primary immunization series and booster immunization was 215 days (135–271) (median (range)). Before the booster (at B0), Spike S1- and RBD-specific memory B cells were detected in 75% and 68% of the participants (Figure 4A,B). A slightly increased percentage of SARS-CoV-2 Spike S1-specific memory B cell-positive participants was noted at B0 compared to 28 days post primary vaccination series (P28) (75% vs. 69%). A paired analysis revealed small differences between P28 and B0 (*p* = not significant (ns) for S1, *p* = 0.02 for RBD) (Figure 4C,D). 

### 3.5. mRNA Booster Immunization Induces a Sharp Increase in Frequencies of SARS-CoV-2-Specific Memory B Cells and Reduces the Overall Number of Low-Responders

Subsequently, the memory B cell response at 28 days after booster immunization (B28) was investigated. A significant increase in the frequencies of both S1- and RBD-specific memory B cells was observed after booster immunization (from 7.8 to 39.5 for S1 and from 5.8 to 38.8 for RBD, *p* < 0.001 for both antigens), indicating that the SARS-CoV-2 booster immunization contributes significantly to the induction of both S1- and RBD-specific memory B cells. More importantly, the percentage of the study participants above the cut-off for specific memory B cells increased from 75% and 68% to 93% for both S1 and RBD after a booster immunization. This included many of the initial low-responders. 

**Figure 5:**
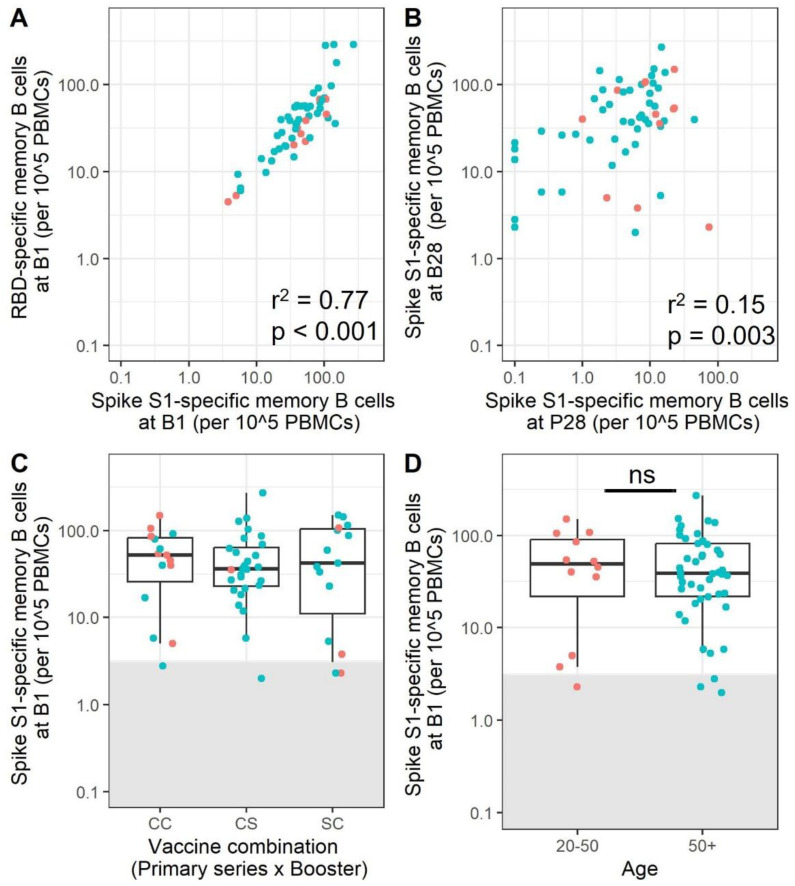
SARS-CoV-2 booster immunization decreases age differences in the memory B cell response. A correlation between Spike S1- and RBD-specific spots at B28 in the same participants is shown for participants positive for both RBD and Spike S1 (N = 54) (**A**). A correlation between SARS-CoV-2 Spike-S1-specific memory B cells at P28 and B28 is shown (N = 58) (**B**) The strength of the correlations was estimated with a Pearson correlation. The SARS-CoV-2 Spike S1 memory B cell response 28 days after completion of the booster immunization was compared between different vaccine combinations (**C**) and between participants split into two different age groups (**D**). (N = 15, 28, and 15 for CCC, CCS, and SSC, respectively (C indicates Comirnaty, S indicates Spikevax)). (N = 12 and N = 46 for the 20–50 and 50+ age groups, respectively). The lower and upper hinges of the boxplot indicate the 25th and 75th percentile, with the middle line indicating the median. The whiskers extend up to 1.5 × the inter-quartile range. Samples within the grey box are below the cut-off for positivity. The strength of the correlation was estimated with a Pearson correlation. Red dots indicate participants aged 20–50, while blue dots indicate participants over 50 years of age. P28 = 28 days after completion of the primary vaccination series; B28 = 28 days after booster immunization; PBMCs = peripheral blood mononuclear cells; RBD = receptor-binding domain; ns = not significant.

Although the booster mRNA immunization induces a notable increase in SARS-CoV-2-specific memory B cells, variation between individuals was still observed. The booster response to Spike S1 correlates well with the response to RBD, as was observed after the primary immunization series (Figure 5A, r^2^ = 0.77, *p* = <0.001). Notably, the majority of the low-responders still present at B28 were part of the lower responders at B0 and B28, although the correlation between memory B cells after primary vaccination and booster vaccination is weak (Figure 5B, r^2^ = 0.15, *p* = 0.003). The combination of mRNA vaccines that was received by the participants did not have a significant influence on the number of SARS-CoV-2 S1-specific B cells after booster immunization (Figure 5C). Most importantly, the difference in the number of SARS-CoV-2-specific memory B-cells previously observed between age groups is no longer apparent after booster immunization (Figure 5D, *p* = 0.7). Therefore, we conclude that booster immunization is essential to increase the memory B cell responses especially in those participants that did not respond very well after the primary vaccinations.

### 3.6. SARS-CoV-2 Omicron BA.1-Specific Memory B Cells Are Present after Immunization

**Figure 6:**
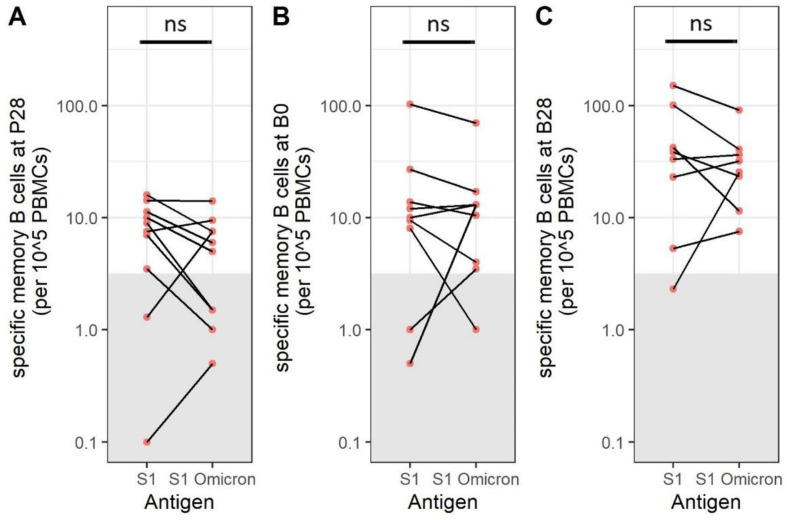
The amount of SARS-CoV-2 Spike S1-specific memory B cells is comparable to SARS-CoV-2 S1 Omicron-specific memory B cells. SARS-CoV-2 Spike S1- and Spike S1 Omicron BA.1-specific memory B cells are compared within the same samples at P28 ((**A**), N = 8), at B0 ((**B**), N = 9), and at B1 ((**C**), N = 8). Samples within the grey box are below the cut-off for positivity. Differences between groups were investigated with a Mann–Whitney U test. Red dots indicate participants aged 20–50, while blue dots indicate participants over 50 years of age. P28 = 28 days after the primary immunization series; B0 = before booster immunization; B28 = 28 days after booster immunization; PBMCs = peripheral blood mononuclear cells; ns = not significant.

It has previously been reported that newly emerged SARS-CoV-2 variants, such as SARS-CoV-2 Omicron BA.1, are less effectively neutralized by antibodies generated against the original strain. Therefore, we also investigated the number of memory B cells targeting SARS-CoV-2 Omicron BA.1 S1 (Figure 6A–C), which showed the same pattern as for Wuhan S1-specific memory B cells, indicating that the presence of cross-reactive B cells is likely. When comparing numbers between the two antigens directly, the difference is not significant, and the effect of the Omicron BA.1 variant tested in the current assay on the number of memory B cells is minimal.

## 4. Discussion

The majority of adults aged 20–90 years of age respond to the SARS-CoV-2 primary vaccination series by producing S1- and/or RBD-specific memory B cells, with the exception of a proportion of predominantly older adults. A third booster vaccination, administered 5–9 months post primary vaccination series, resulted in a large increase in the frequencies of S1- and RBD-specific memory B cells in almost all participants. 

The literature describing SARS-CoV-2-specific memory B cells after immunization is often limited to adults up to 65 years of age, where a stable memory B cell pool is observed after the primary immunization series [9], followed by a further increase in the memory B cell frequency and repertoire diversity after booster immunization [28,29,30,31]. Reduced memory B cell frequencies were previously observed in older adults over 70 years of age when compared to younger adults after a first vaccination [32]. These differences were no longer apparent between age groups after a second vaccination. Our study identified a heterogeneous pattern of memory B cell responses in older adult participants over 50 years of age, with a subgroup responding to the first two vaccinations with very low numbers of memory B cells.

A low memory B cell response to immunization has previously described in older adults for several pathogens, such as influenza and *Bordetella pertussis* [16,33]. In contrast to the current study, the majority of immunizations in older adults induce a recall response. In such cases, the reduced responses in older adults have been ascribed to changes in B cell subset composition, or a reduced ability to differentiate memory B cells into antibody-producing plasma cells [13,34,35,36]. Low numbers of naïve B cells have been described in older adults, potentially reducing the ability of the B cell response to generate diverse antibody responses to new antigens [13]. In addition, it has been reported that SARS-CoV-2-specific total T cell or CD4^+^ T cell responses to SARS-CoV-2 vaccination are reduced in older adults [37,38]. These factors may partially explain the low memory B cell response in a subset of adults in the current study. There are several groups of vulnerable people in the general population who do not respond well after the primary SARS-CoV-2 immunization series, such as transplant patients or individuals taking immunosuppressive medication [39,40]. These responses resemble those of the low-responders in our cohort, though the majority of them did not report any clear comorbidity that could explain the reduced vaccination response we observed. Reduced naïve CD4^+^ T cells and peripheral dendritic cells have previously been associated with reduced vaccine responses in older adults after vaccination with a live-attenuated yellow fever vaccine [41]. Therefore, further phenotyping of B cell, T cell, and dendritic cell populations is needed to substantiate our findings and to further investigate differences between responders and low-responders.

One of the limitations of our study relates to the roll-out of the national immunization program in the Netherlands. Older and vulnerable populations received their primary immunization earlier compared to young participants, resulting in 4–9-month intervals between primary and booster immunizations, with longer intervals primarily occurring in the older age group. For some participants, there seemed to be an increase in specific memory B cells just before the booster immunization compared to 28 days after completion of the primary series. It has been reported that germinal centers induced by mRNA vaccines can still be detected after 6 months, potentially contributing to maintaining or increasing the memory B cell response [42] Importantly, no correlation was observed between the SARS-CoV-2 Spike S1-specific memory B cells present after booster immunization and vaccine interval. An interval of 4–9 months between the primary immunization series and the first booster immunization resulted in good memory B cell responses, even in older participants in the study. The interval between the two primary immunizations in our study was 35 days (28–48 (median(range)) for participants at P28. While it has been reported that a longer interval between the two primary immunization results in an increase in antibody responses and a reduction in T cell responses [43,44,45], it is not clear how this would have impacted the memory B cell response measured in the current study. 

There is not yet an official correlate of protection against SARS-CoV-2 infection or COVID-19 hospitalization, making it difficult to determine what qualifies as a good immune response. In addition, the constantly changing variants of concern (VOC) further complicate our understanding of protection [3,29,46]. Within the small subgroup in which we compared the response to the original S1 and the Omicron BA.1 S1, no strong deviations in the number of specific memory B cells were observed, suggesting a large degree of cross-reactivity between the two variants. However, memory B cells generated through immunization may induce less Omicron BA.1-specific neutralizing antibodies compared to neutralization towards the vaccine strain, and further investigation into this subject would be beneficial for our understanding of the SARS-CoV-2-specific immune response.

In conclusion, our data show that memory B cell populations seem stable during the initial months after primary immunization, as has been reported by others, indicating that the memory B cells induces by this vaccine may be relatively long-lived [9]. In addition, we show that a booster immunization is important for the generation of a memory B cell response in those older adults showing a suboptimal response to the primary SARS-CoV-2 immunization series. This 3rd mRNA vaccine dose clearly stimulated the SARS-CoV-2 memory B-cell pool in most participants. It is anticipated that the specific memory B cell pool will play a significant role in the recall response following viral (re-)exposure, which is considered instrumental in providing immune protection. Although repeat immunizations may be essential for vulnerable populations, it may not be required as frequently for the good responders in the older population in our study. Extended studies investigating the stability and quality of SARS-CoV-2-specific memory B cells will be required to learn more about their role in long-term protection.

## Figures and Tables

**Table 1:** Participant Characteristics. For the vaccine combination, C represents Comirnaty and S represent Spikevax. P28 = 28 days after completion of the primary immunization series. B0 = just before booster immunization; B28 is 28 days after booster immunization. For the vaccine interval, primary interval is the interval between the two immunizations that are part of the primary immunization and the booster interval is the interval between the last primary immunization and the first booster immunization.

	All Participants	Adults (18–50)	Older Adults (50+)
size (N	88	26	62
age (median (range))	66 (20–99)	32.5 (20–49)	71 (52–99)
sex (N(% female))	52 (59)	16 (62)	36 (58)
Primary immunization			
Comirnaty (N (%))	59 (68)	17 (65)	43 (69)
Vaccine combination			
CCC (N (%))	15 (26)	8 (67)	7 (15)
CCS (N ( %))	28 (48)	1 (8)	27 (59)
SSC (N (%))	15 (26)	3 (25)	12 (26)
Sample availability			
P28 (N (%))	87 (99)	26 (100)	61 (98)
B0 (N (%))	44 (50)	6 (23)	38 (61)
B28 (N (%))	58 (66)	12 (46)	46 (74)
Vaccine Interval (days)			
Primary (median (range))	35 (28–48)	35 (28–48)	35 (28–42)
Booster (median (range))	215 (135–271)	157 (140–225)	218 (135–271)

## Data Availability

Full data will be made available upon reasonable request.

## Supplementary Materials

The following supporting information can be downloaded at: https://www.mdpi.com/article/10.3390/vaccines11071196/s1, Figure S1: Cut-off establishment for low-responders using the MERS-S1 antigen; Figure S2: Gating strategy for the identification of plasmablasts and memory B cells; Figure S3: RBD-specific memory B cells after primary immunization; Figure S4: Differences in numbers of memory B cells and plasma cells between responders and low-responders after proliferation ex vivo; Figure S5: Spike S1-specific memory B cells adjusted for percentages of plasmablasts and memory B cells; Figure S6: No evidence for effects of vaccine interval on memory B cell response; Table S1: B cell subset percentages before and after culture.
